# Diel periodicity of *Drosophila suzukii* (Diptera: Drosophilidae) under field conditions

**DOI:** 10.1371/journal.pone.0171718

**Published:** 2017-02-10

**Authors:** Richard K. Evans, Michael D. Toews, Ashfaq A. Sial

**Affiliations:** Department of Entomology, College of Agricultural and Environmental Sciences, University of Georgia, Athens, Georgia, United States of America; University of Mississippi, UNITED STATES

## Abstract

*Drosophila suzukii* Matsumura (Diptera: Drosophilidae), an economically important pest of blueberry and other thin-skinned fruits, persists and prolifically reproduces under seemingly lethal climatic conditions in the field. However, behavioral and physiological mechanisms employed by *D*. *suzukii* to tolerate such extreme climatic conditions in the field are unknown. The primary objective of this project was to investigate diel periodicity of *D*. *suzukii* and their reproductive success under field conditions as related by climatic factors such as temperature and relative humidity. Results show that *D*. *suzukii* reproductive success was significantly higher during the night (including dawn and dusk periods) than the day in terms of oviposition, pupation, adult eclosion, and the number of progeny per female. Female *D*. *suzukii* reproductive success was not significantly different between specific regions of a blueberry bush in relation to the amount of shade provided by the canopy. Our studies indicate that *D*. *suzukii* flight activity is crepuscular and is sensitive to fluctuations in temperature and relative humidity. Results also suggest that the majority of fly activity during peak hours is concentrated in areas around the border and within the center of blueberry orchards with little activity in the surrounding wooded areas. These findings suggest that *D*. *suzukii* prefers microclimate with mild temperatures and high humidity, and does not function well when exposed to direct sunlight with extreme heat. The authors propose that *D*. *suzukii* management strategies should be implemented during the early morning and immediately before darkness to maximize efficacy.

## Introduction

*Drosophila suzukii* Matsumura (Diptera: Drosophilidae) is one of the most serious pests of thin-skinned fruits including blueberry, blackberry, cherry, raspberry, and strawberry [1, 2, 3, 4]. The propensity for *D*. *suzukii* to prefer ripening fruit makes it a particularly difficult pest for growers to deal with. Female *D*. *suzukii* possess a serrated ovipositor that enables oviposition in fruit that is ripening or undamaged [3,4], as opposed to other members of the Drosophilidae that oviposit into overripe or previously damaged fruit [2]. Developing larvae consume the flesh of the fruit causing it to become soft and rot rapidly, making the fruit unmarketable for fresh consumption. This results in reduced crop yields and significant financial losses which have been estimated at $718 million annually in the United States [1, 2, 5].

The economic impact of *D*. *suzukii* infestation is enormous for small and stone fruit producers with decreases in revenue reaching 30–40% [5]. Additionally, the cost of treating *D*. *suzukii* is both economically and environmentally prohibitive. Current management programs consist of preventative applications of broad-spectrum insecticides including organophosphates, pyrethroids, carbamates, and spinosyns to protect fruit from *D*. *suzukii* infestation [1, 6]. Broad-spectrum insecticide applications eliminate beneficial insects and result in secondary pest outbreaks that require additional inputs. Repeated insecticide applications also result in insecticide resistance in *D*. *suzukii* [7] while simultaneously increasing the risk of rejection of the fruit in local or export markets due to insecticide residue issues. It is therefore of paramount importance to develop strategies to reduce the number of applications to control *D*. *suzukii* which may not be possible without clear understanding of biology and behavior of *D*. *suzukii* in the field.

Plastic cup traps baited with apple cider vinegar or a yeast-sugar-water solution are commonly used to monitor *D*. *suzukii* presence in the field [8]. However, captures in these traps do not correlate well with the observed fruit infestation and therefore cannot be used to trigger management interventions (Burrack et al 2015). In the absence of good economic thresholds, growers currently utilize prophylactic insecticide applications. These control programs can be improved by better understanding the effect of climatic factors such as temperature, relative humidity, and diel periodicity of *D*. *suzukii* activity under field conditions [9, 10].

When exposed to temperatures above their optimal developmental threshold of 24–26°C, *D*. *suzukii* and other members of the Drosophilidae display a decrease in reproductive and developmental success [8, 10]. However, majority of the fruit growing regions routinely experiences high temperatures (32–38°C) during hottest part of the day in the fruit harvest season, yet growers report fruit *D*. *suzukii* infestations. The physiological mechanisms and behavioral adaptations enabling *D*. *suzukii* to survive these lethal temperatures in the field are largely unknown. A recent study indicated that members of the Drosophilidae were more heat tolerant at high relative humidity (>70%) [11]. Hence, it is possible that increased relative humidity may mitigate the detrimental effects of high temperatures on *D*. *suzukii* reproduction. The actual impact of humidity on the ability of *D*. *suzukii* to survive potentially lethal temperatures remains to be investigated.

Daily activity rhythms are one of many physiological processes that are governed by the circadian clock [12]. Both physiological and abiotic factors entrain the circadian clock in insects, commonly referred to as zeitgebers. Two of the most important zeitgebers for entraining the circadian clock are temperature and light. Each of these factors can function independently, but integration of the two factors occurs under outdoor conditions and serves to coordinate the activity rhythms [13, 14]. A previous study showed that *D*. *melanogaster* displayed bimodal activity peaks under laboratory conditions whereby locomotor activity increased in the morning (lights on) and evening (lights off) [15]. Similarly, in a laboratory-based study, *D*. *suzukii* displayed a bimodal activity profile under simulated natural “summer” conditions, which is attributed to extended thermophases associated with longer summer days [16]. Observed daily activity is regulated by the presence of a circadian clock. The concept of the circadian clock governing daily activity is well understood in *D*. *melanogaster*, but the chronobiology and daily rhythms of *D*. *suzukii* under the field conditions have not yet been investigated. Chronobiology, as it relates to daily activity patterns of *D*. *suzukii*, could provide valuable information to align the timing of the implementation of control strategies with the peak fly activity during a 24-hour period.

The objective of this project is to explore chronobiology of *D*. *suzukii* under field conditions with regards to how daily rhythms as well as the development and reproduction are affected by temperature and relative humidity during a 24-hour cycle [17, 18]. Our findings will not only reveal daily activity patterns but also elucidate reproductive success of *D*. *suzukii* under natural field conditions. Results will help farmers to predict relative pressure from *D*. *suzukii* in their crops, and can be used as a reproductive physiology-based comparative risk tool to predict larval infestation in a particular field [19, 20, 21, 22, 23, 24]. Additionally, fruit producers will be able to utilize this information to apply control strategies during the peak activity periods of *D*. *suzukii* which will significantly improve effectiveness of the current control strategies leading to fewer insecticide applications needed to control *D*. *suzukii*.

## 2 Materials and methods

### 2.1 Insect rearing and treatment groups

*D*. *suzukii* used in this study were obtained through a laboratory colony established from approximately 500 wild specimens captured in Clarke County, GA, during the summer of 2013. Wild *D*. *suzukii* were periodically added towards the end of each successive summer to maintain genetic variability in the lab colonies. The fly colony was reared on standard fly diet (65.1 g cornmeal, 13.0 g yeast, 6.8 g agar, 55.0 ml molasses, 14.5 ml tegosept, 2.4 g propionic acid salt, per 1.1 liter dH2O) as described in Emiljanowicz et al. (2014). Tegosept and propionic acid were used to slow the growth of fungus/mold in the rearing bottles [20]. Fly cultures were maintained in 177-ml square bottom polypropylene bottles (Genesee Scientific, San Diego, CA) with 50 ml of fly diet and plugged bonded dense-weave cellulose acetate plugs. Incubators (Model I36VLCB, Percival Scientific, Perry, IA) set to 22C, 70% relative humidity, and a photoperiod of 13:11 (L:D) h were utilized for colony maintenance. The photoperiod, temperature, and relative humidity were set at thresholds which mimic the ambient field conditions *D*. *suzukii* is exposed to in southeast Georgia. *D*. *suzukii* adults aged 5–7 days were used for all experiments.

### 2.2 Reproductive success of *D*. *suzukii* under field conditions

#### 2.2.1 Field cage study

**Fig 1:**
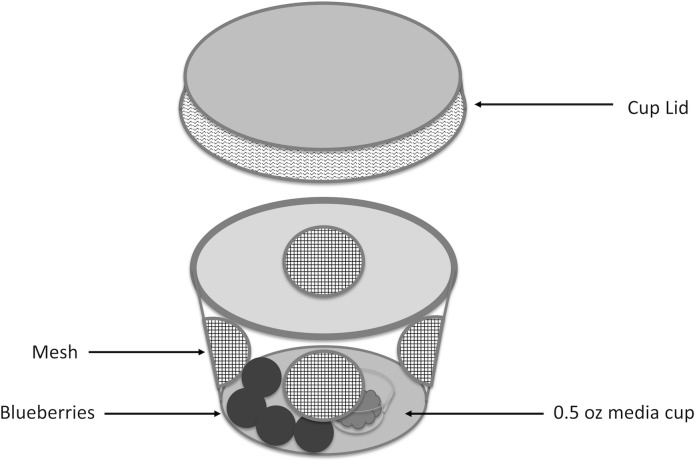
A schematic diagram of field cages used in the field cage study.

This study was conducted at the Blueberry Research and Demonstration Farm located in Alma, GA (Bacon County, 31°32'03.8"N 82°30'36.5"W). The orchard contained 5 year old Rabbiteye blueberries of the powder blue variety. No pesticides were applied in the experimental block to avoid confounding observations. The field cages for this study were designed using protocols from Overgaard et al. [10]. The cages used for the treatment groups were clear 2 oz. (Fabri-Kal, Kalamazoo, MI) plastic soufflé cups containing 4 fresh blueberries (previously scanned under a microscope to ensure no oviposition had occurred) as an oviposition substrate and approximately 2 ml of diet as an additional source of moisture. The fly diet was contained in 0.5 oz cups (Fabri-Kal, Kalamazoo, MI) affixed inside the larger 2 oz cup. The smaller cup was glued to the center within the larger cup providing both a structural support for the blueberries and preventing potential movement inside the assay chamber that could crush test subjects. Four equidistant holes along the sides of the cup were covered with lightweight mesh netting to allow the climate inside the cup to be as similar to natural conditions as possible, while also keeping the individuals confined. Four randomly selected 5–7 d-old adult *D*. *suzukii* (2 males and 2 females) were placed in each cage. Cages placed inside the canopy were fashioned with a small wire hook allowing for the cup to be placed directly in the middle of the bush (Fig 1).

Field cages were placed at three different locations in the blueberry bushes based on the level of canopy coverage (shade). The three locations were as follows: “between”—space between two bushes (half sun-half shade); “outside”—outside of a row of bushes (all sun); “inside”—inside the canopy of a bush (all shade). These areas were selected to determine habitats that can support *D*. *suzukii* during periods of extreme heat, where temperatures are routinely within the range of 30–37°C during the day. A 24-hour day was split into two 12-hour periods, including “day” 8 a.m.- 8 p.m. and “night” 8 p.m.– 8 a.m. The split was done to compare *D*. *suzukii* reproductive success during night and day. The night period included the majority of both dawn and dusk environments centered on civil twilight. The dawn period included sunrise (6 a.m.) and civil twilight (6:40 a.m.). Sunset begins at 7 p.m. with civil twilight occurring for dusk near 8 p.m., with dusk essentially occurring between 8:45–9:00 p.m. Both of these times were included in the night period. The day period was setup to include the largest amounts of both light and heat stress *D*. *suzukii* would be exposed to. Thus we have setup the day:night split so the night period includes the specific hours of civil twilight within dawn and dusk when the authors propose the majority of fly activity occurs. Accompanying each location was a HOBO logger (Onset, Clayton, NC), either Pro v2 U23-003 or U12-012 data loggers for measurement of temperature and humidity readings every 10 minutes (S1 Table).

Cages were left in the field for 12 hours and afterward taken from each location to the laboratory where oviposition counts were completed. The process was repeated every 12 hours for a week, which resulted in a total of 30 replicates for each location per time of day. Following the oviposition in the field, eggs were allowed up to 2–3 weeks to develop. The infested berries were dissected and the number of pupae and adults was recorded. The number of progeny per female fly was measured by dividing the number of adult flies that eclosed by the number of female flies in each cup. The same method was used for calculating number of eggs laid per female. The number of larvae to hatch from eggs were not included in the present study due to the need of destructive sampling. This type of sampling might prevent the larvae from reaching the adult stage. The percent pupation and the percent adult eclosion were calculated by dividing the number of pupae or adults observed from each cup by the number of eggs that were laid. Data on survivorship, development, and reproductive parameters were used to determine *D*. *suzukii* reproductive success under natural field conditions in a blueberry orchard.

#### 2.2.2 Field infestation study

The experiment was designed to measure the impact of location under the canopy and time of the day on *D*. *suzukii* survivorship, development, and reproductive parameters under natural field conditions in a blueberry orchard. The study was conducted at the same blueberry orchard located in Alma, GA (Bacon County, Georgia 31°32'03.8"N 82°30'36.5"W). The orchard contained 5 years old Rabbiteye blueberries of the powder blue variety. No pesticides were applied in the experimental block to avoid any negative effects of pesticides on activity and survivorship of *D*. *suzukii*. In order to test the difference between *D*. *suzukii* reproductive success from natural infestation in the field and a controlled laboratory environment, netted chambers were placed at three different locations on a blueberry bush in relation to canopy coverage and sunlight present. The blueberry bush was therefore divided into microhabitats similar to what was described in the field cage study mentioned above. Subsequently, these three chamber locations in blueberry canopy will be referred to as ‘inside’, ‘outside’, and ‘between’. In addition to microhabitats, reproductive parameters were compared by night and day using the same day:night setup described above. Furthermore, it is important to note that this experiment was carried out under the constant threat of thunderstorms developing, data were only included from days/nights not under stormy conditions to avoid weather as a confounding factor.

**Fig 2:**
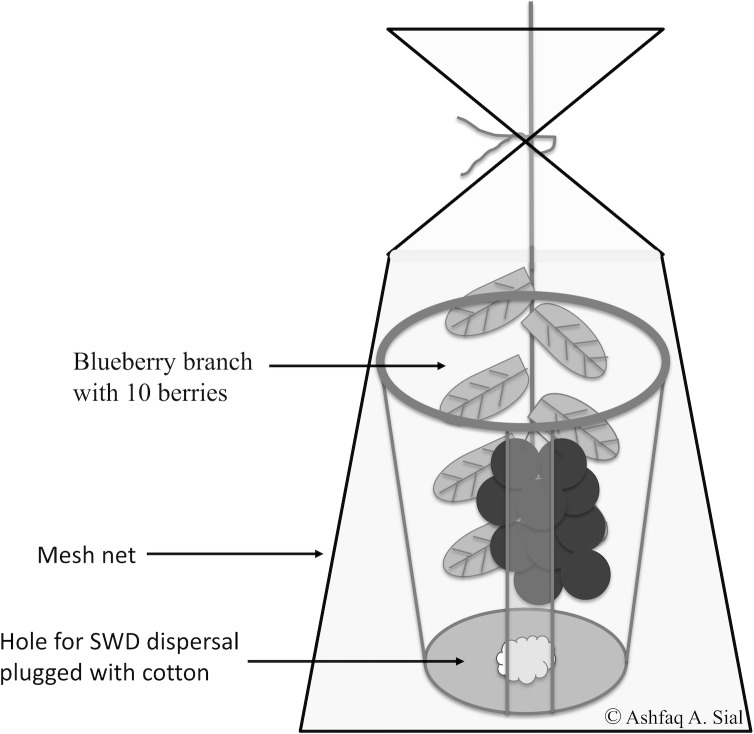
A schematic diagram of field chambers used in the field infestation study.

For this experiment, we used a field chamber outlined with only a thin-transparent net. A set of ten 5–7 d-old adult *D*. *suzukii* were added to each chamber (5 male, 5 female). Chambers were developed by excising the majority of 32 oz. clear plastic PET (Fabri-Kal, Raleigh, NC) containers, two small columns of plastic (2-3cm in width) were left attached to the top and bottom of the cup to provide structural support for the chamber. Thin mesh netting was then attached via hot glue to the remaining portion of the cup, leaving enough loose netting to allow the container to be bound onto a blueberry bush. Chambers were attached to a blueberry branch containing exactly 10 blueberries located in each of the three different microhabitats. These blueberries were inspected for the presence of previous oviposition, only berries with no previous oviposition were used. A small hole, approximately 3 cm. in diameter, was cut into the base of the chamber to allow for dispersal of lab colony *D*. *suzukii* into the chamber. This design prevents test subjects from escaping while maintaining natural conditions inside the chamber (Fig 2). A total of five chambers were deployed for each treatment group for a total of 15 replicates for each treatment. HOBO Data loggers model U12-012 (Onset, Clayton, NC) accompanied each chamber to record temperature and relative humidity every 10 minutes for the experiment’s duration. Chambers were collected following each period and adult survival was noted and oviposition was observed via dissecting microscopy. The number of eggs laid per female, percent pupation, percent adult eclosion, and progeny per female were analyzed by the effects of location and time of the day (S2 Table).

### 2.3 Fly activity based on time of the day and microclimate

2015 Study: This study was conducted during the summer of 2015 at a blueberry orchard located in Brantley County, Georgia (31°10'58.2"N 82°09'57.7"W), then replicated during the same season at an orchard located in Bacon County, GA (31°30'41.9"N 82°27'28.4"W). The Brantley County orchard contained eight years old southern highbush blueberries of star variety and the Bacon County orchard contained seven years old rabbiteye blueberries of powder blue variety which were adjacent to wooded areas. No pesticides were applied in the experimental block to avoid any negative effects of pesticides on activity and survivorship of *D*. *suzukii*.

To determine where in a blueberry orchard *D*. *suzukii* is most active, the orchard was divided into three different locations or microclimates. Microclimates were selected based on the amount of sunlight the area receives during a clear 24-hour day. The three locations were: (1) the “woods” adjacent to the orchard; (2) the “border” of the unmanaged field; and (3) the “field” which was located near the middle of the orchard. A set of three 32-oz plastic cup traps baited with drowning solution containing a sugar-yeast-water slurry was placed in each of the three microclimates. The mixture was comprised of 135g sugar, 30g yeast, 1ml detergent, and 150ml tap water was kept in 32oz containers (PFS Sales Co., Raleigh, NC). The mixture was designed to kill any visitors to the trap via drowning solution. Containers were covered with a lid and metal wire. Holes were soldered approximately 2 cm in diameter near the mouth of the container for *D*. *suzukii* entry. Traps were strategically placed in the center of the canopy of blueberry bushes at the field and border locations. Traps within the woods were hung on branches near dense foliage.

**Fig 3:**
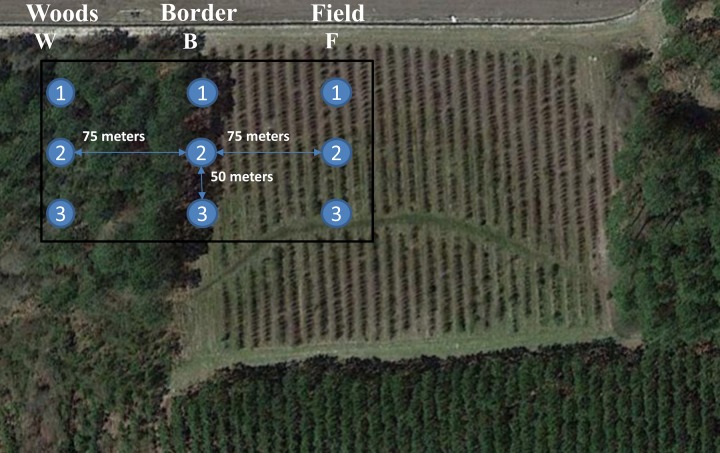
Satellite imagery of the blueberry orchard in Bacon County, GA shows orchard layout with trap location and relative distance between each trap. Traps were spaced 75 meters apart between each location and 50 meters apart within each location.

Each microclimate had a total of three baited traps placed no less than 50 m from each other. Traps between different locations were given a 75 m buffer zone to minimize interactions from adjacent locations. Fig 3 shows a satellite image of each location with trap setup overlay.

In order to determine *D*. *suzukii* activity during different times of the day, within each microclimate/location, the 24-hour day was divided into four 6-hour periods with distinct climatic conditions. The 6-hour periods were described as dawn (5:30am—11:30am), day (11:30am—5:30pm), dusk (5:30pm– 11:30pm), and night (11:30pm– 5:30am). Each period represents a different position of the sun which provides a distinct microclimate based on time of the day and location.

A total of 36 baited traps were used during 24-hour period such that a subset of 9 traps was deployed for each of the 6-hour periods. Baited traps were replaced at the end of each 6-hour period. Between each period the traps were not replenished but stored in re-sealable plastic bags and reused at the appropriate 6-hour intervals. However, traps were supplemented with fresh bait solution to mitigate the change in chemical composition over time. This activity was repeated daily for a week. At the end of the week, the number flies caught in each trap was recorded for each microclimate giving a quantitative measure for *D*. *suzukii* activity during each of the 6-hour periods within a 24-hour day.

In addition to trap counts, observations were recorded for temperature and relative humidity within the three microclimates during each 6-hour period using HOBO Pro v2 (ONSET) data loggers. Temperature and humidity measurements were taken every 10 min to achieve dynamic measurement of the constantly changing climatic conditions for each microclimate (S3 Table, S4 Table). At the end of the week, all traps were collected and the number flies caught per trap was recorded for each microclimate, giving a quantitative measure for differences in *D*. *suzukii* activity by both location and during each of the 6-hour periods within a 24-hour day.

2016 Study: The study was replicated in 2016 at an orchard in Bacon County, GA (31°30'41.9"N 82°27'28.4"W). The orchard used contained eight years old rabbiteye blueberries of the powder blue variety which were adjacent to wooded areas. No pesticides were applied in the experimental block to avoid any negative effects of pesticides on activity and survivorship of *D*. *suzukii*. The experimental protocols were similar to those used in 2015 study. A slight change was made in 2016 with respect to checking the traps daily instead of weekly to observe for any change in possible attractiveness over time based on the number of flies recorded.

### 2.4 Data analysis

In the fly activity by time of day experiment, trap was the experimental unit and number of fly captures per trap was the response variable. For the 2015 data, this experiment was organized as a two-way factorial arrangement of treatments in a randomized complete block design, with time of day and location of cage as the fixed effects and farm where the experiment was conducted serving as the blocking variable. For the 2016 data, all data were collected at the same farm so the experimental design was simply a completely randomized design. Ratio of females to males was analyzed by dropping all data points with zero captures and then conducting the analyses on ratio of females divided by total number of captures in that trap. In the field cage study, the experimental unit was the cage provisioned with blueberries and two females. This experiment was organized as a two-way factorial arrangement of treatments in completely randomized design, with time of day and location of the cage in or near the plant canopy as the fixed effects. In that specific experiment, data on progeny survival were analyzed as a three-way factorial arrangement of treatments (fixed effects included fly gender, time of day and location of cage) in a completely randomized design. The field infestation study was organized identical to the field cage study, except that data on progeny survival were not scored.

For the field cage and field infestation experiments, the number of eggs laid per female was calculated by dividing the number of eggs observed from each treatment group by the number of females in the group (5). After 2–3 weeks, the pupal and total adult presence were recorded to provide an indication of the percentage of fertile eggs laid. Percent pupation was measured by dividing the number of pupae by the number of eggs laid. The percent adult eclosion was measured by dividing the number of adults by the number of eggs laid. Progeny per female were calculated by dividing the number of adults by the number of female parents (5) for that treatment group.

Experiments were analyzed using Generalized Linear Mixed Model Analyses [PROC GLIMMIX, SAS 2013]. Fixed effects were considered significantly different at the alpha = 0.05 level, while interactions were considered significant at the alpha = 0.1 level. Increasing the significance level for interactions enabled the authors to characterize nuance of how the measured responses changed at different levels of each main effect, which contributes to a better understanding insect behavior in the field. When two-way interactions were detected, independent fixed effects were not examined in favor of examining the interaction using the slice option (SAS 2014); this procedure allows the investigator to make main effect comparisons (at the alpha = 0.05 level) within a fixed level of the interactive factor. Comparisons among qualitative variables with a significant F-test were conducted using pairwise comparisons. Response variables that were collected as proportions or percentage were arcsine transformed [25] when the transformation improved model fit as assessed using Pearson residual plots. Means and associated measures of variability were back transformed for data presentation.

## 3 Results

### 3.1 Reproductive success of *D. suzukii* under field conditions

#### 3.1.1 Field cage study

**Fig 4:**
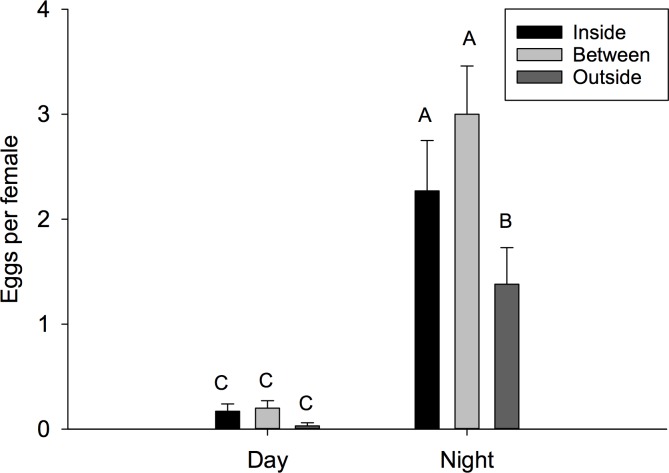
Mean number of eggs per female. Mean number of eggs per female (± SEM) oviposited during the day or night in field cages placed inside the plant canopy (full shade), between adjacent bushes (partial shade), or completely outside the plant canopy (no shade). Different letters signify statistical differences among means (LSMEANS test).

**Fig 5:**
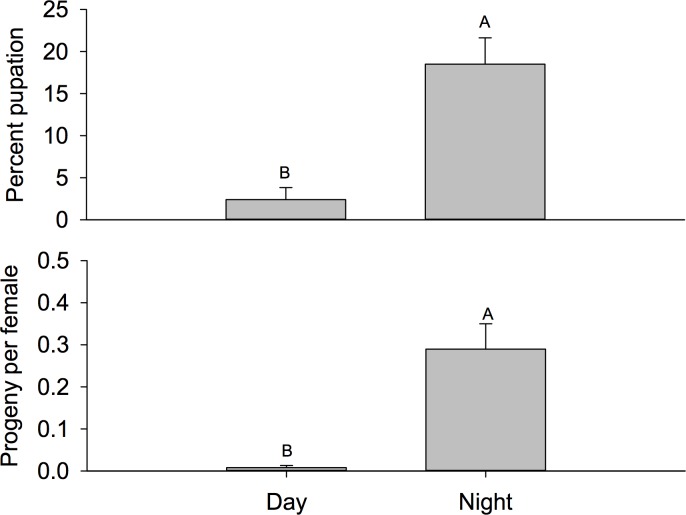
Percent pupation. Percent pupation (± SEM) of flies caged during the 12-hour periods designated as day or night including dawn and dusk (top), and F1 progeny per female (± SEM) during the day or night (bottom). Different letters signify statistical differences among means (LSMEANS test).

Number of eggs per female, percent pupation and number of adult progeny were analyzed. There was a significant time by location interaction for number of eggs per female (F = 2.66; *df* = 2, 174; P = 0.0725). When examining this interaction using the slice option, we found that there were no differences among locations during the day (F = 0.08; *df* = 2, 174; P = 0.9242), but there were significant differences at night (F = 6.64; *df* = 2, 174; P = 0.0017) (Fig 4). There were fewer eggs per female in cages located outside the plant canopy compared to cages located either between bushes in inside the canopy. For example, more than two times the number of eggs per female were observed between plants compared to those located completely outside the canopy. Conversely, there was not a significant interaction for percent pupation (F = 1.16; *df* = 2, 174; P = 0.3172). The main effect test for location was not significant (2.43; *df* = 2, 174; P = 0.0913), but there was a significant difference between night and day (F = 22.24; *df* = 1, 174; P < 0.001) (Fig 5). Greater than 7-fold more pupae eclosed from eggs laid at night compared to during the day. Similarly, there was not an interaction observed for progeny per female (F = 0.59; *df* = 2, 174; P = 0.5528) or the main effect for location (F = 0.66; *df* = 2, 174; P = 0.5159), but significantly more progeny per female were observed for cages present during the night (F = 21.27; *df* = 1, 174; P <0.0001).

**Fig 6:**
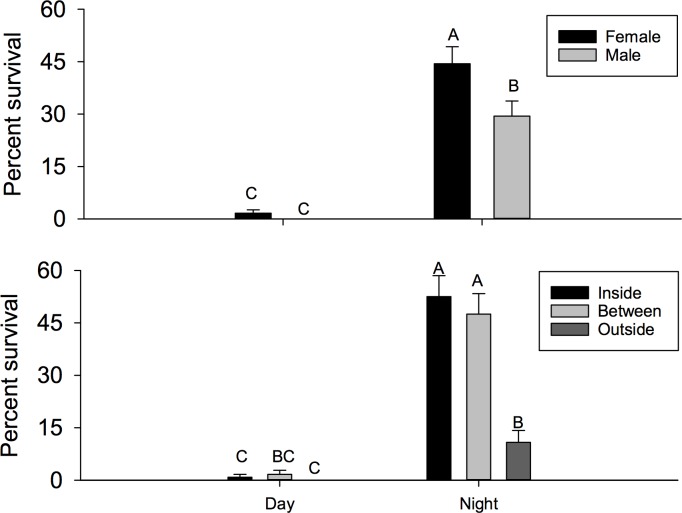
Mean percent survival. Mean percent survival (± SEM) by time of day and gender (top) and mean percent survival (± SEM) by time of day and field cage location (bottom). Different letters signify statistical differences among means (LSMEANS test).

When comparing differences in percent fly survival among the fixed effects including gender, location and time, there was not a three way interaction (F = 0.18; *df* = 2, 348; P = 0.8373), but two of the two-way interactions were significant. The tests for time by gender (F = 4.87; *df* = 1, 348; P = 0.0278) and time by location (F = 17.82; *df* = 2, 348; P < 0.0001) (Fig 6) both showed significance. These interactions occurred because there were virtually no flies that survived in cages deployed during the day. Conversely, approximately 30% of the males survived at night compared with 45% of females at night (Fig 6). Similarly, across gender and time, there was increased survival for flies deployed either inside or between canopy locations compared to outside the canopy (F = 20.06; *df* = 2, 348; P <0.001).

Temperature and relative humidity data were collected using HOBO data loggers placed in each location. For this experiment, the relative humidity at night (94.74 ± 0.41) was significantly higher than the day (56.51 ± 0.39) (*F* = 4486.91, *p* <0.0001, *df* = 1). However, it was not significantly different at different locations within the canopy (*F* = 2.08, *p* = 0.1251, *df* = 2). The temperature at night (24.1 ± 0.17°C) was significantly lower than the day (33.9 ± 0.16°C) (*F* = 5570.57, *p* <0.0001, *df* = 1). The mean temperature for the ‘outside’ location (29.7 ± 0.37°C) was significantly higher than the ‘between’ location (28.8 ± 0.37°C) (*F* = 7.78, *p* = 0.0088, *df* = 2). The ‘inside’ location (29.1 ± 0.36°C) was not statistically different from either ‘outside’ or ‘between’ locations.

#### 3.1.2 Field infestation study

**Fig 7:**
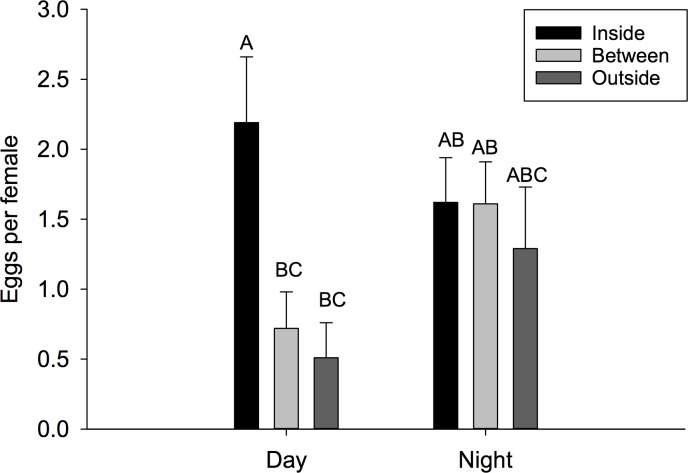
Mean number of eggs per female (± SEM) by time of day and chamber location. Different letters signify statistical differences among means (LSMEANS test).

**Fig 8:**
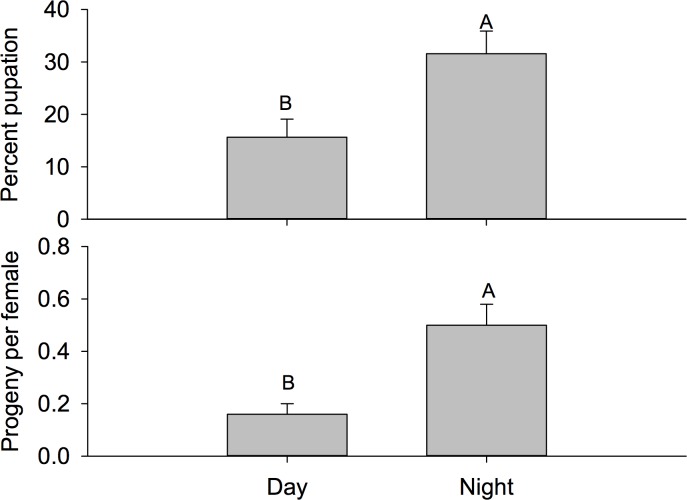
Mean percent pupation. Mean percent pupation (± SEM) by time of day (top) and mean progeny per female (± SEM) by time of day (bottom). Different letters signify statistical differences among means (LSMEANS test).

Generally speaking the trends in this study were very similar to those observed in the field cage study, however greater oviposition was observed in this experiment overall. There was a significant time by location interaction for number of eggs per female (F = 2.65; *df* = 2, 84; P = 0.0762). When examining this interaction using the slice option, we found that there were differences among locations during the day (F = 6.80; *df* = 2, 84; P = 0.0018), but there were no differences at night (F = 0.30; *df* = 2, 84; P = 0.7406) (Fig 7). There were similar numbers of eggs per female among locations at night; however, four-fold fewer eggs per female were oviposited outside the canopy at night compared to inside the canopy at night. During the day, the number of eggs per female in chambers located between the canopy of two bushes were similar to outside the canopy. Conversely, there was not a significant interaction for percent pupation (F = 1.47; *df* = 2, 84; P = 0.2365). The main effect test for location was not significant (1.22; *df* = 2, 84; P = 0.3013), but there was a significant difference between night and day (F = 8.47; *df* = 1, 84; P < 0.0046) (Fig 8). Twice as many pupae eclosed at night compared to during the day. Similarly, there was not an interaction observed for progeny per female (F = 1.94; *df* = 2, 84; P = 0.1508) or the main effect for location (F = 2.51; *df* = 2, 84; P = 0.0876), but significantly more progeny per female were observed for cages present during the night (F = 14.42; *df* = 1, 82; P <0.0003).

The relative humidity was significantly higher at night (93.83 ± 0.44) than the day (72.63 ± 0.49) (*F* = 999.61 *p* <0.0001, *df* = 1). In relation to location within the blueberry canopy relative humidity was significantly different (*F* = 11.47 *p* <0.0001, *df* = 2). The mean relative humidity was significantly higher under the canopy (‘inside’ location) of the bush (86.20 ± 0.71) than outside the bush (82.78 ± 0.71). The ‘between’ the bush location was not statistically different (84.69 ± 0.71) from either the ‘outside’ or ‘inside’ locations. When compared by time of the day, the mean temperature at night (22.79 ± 0.17°C) was significantly lower than the day (28.9 ± 0.20°C) (*F* = 1652.6 *p* <0.0001, *df* = 1)). However, temperature was not statistically different between locations (*F* = 1.35 *p* = 0.2595, *df* = 2). The mean temperature for all three chamber locations was 25.3–25.7°C. The interaction between location and time of day on temperature was significant at each level (*F* = 5.82 *p* = 0.0030, *df* = 2), the interaction shows that differences in temperature by location is dependent on time of day.

### 3.2 Fly activity based on time of the day and microclimate

**Fig 9:**
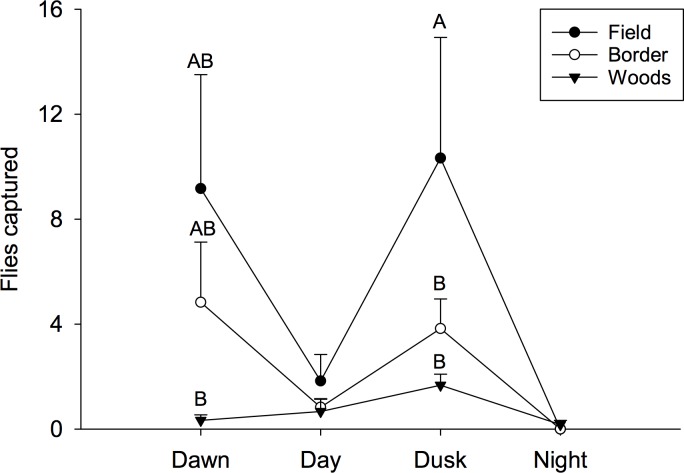
Mean number of spotted wing drosophila captured at different times of day and locations near the orchard in 2015. Different letters signify statistical differences among locations within times of day (LSMEANS test).

Number of observed fly captures varied by habitat and time of day. The test for an interaction between time of day and microclimate was significant (F = 1.90; *df* = 6, 59; P = 0.0954). Further examination of this interaction broken down by time of day, showed that there were profound differences among habitats during two of the four times of day (Fig 9). Regardless of time of day, there were never more than 1.5 captures in the wooded habitat. Conversely, there were greater than nine-fold more flies captured in the field during the dawn and dusk period compared to the same time periods in the woods (F = 6.08; *df* = 2, 59; P = 0.0040). The mean number of flies in the border habitat was approximately half of those recorded at peak flight times in the field (F = 6.34; 2, 59; 0.0032). No differences in fly captures were detected during the day and zero fly captures were recorded at night in all habitats.

**Fig 10:**
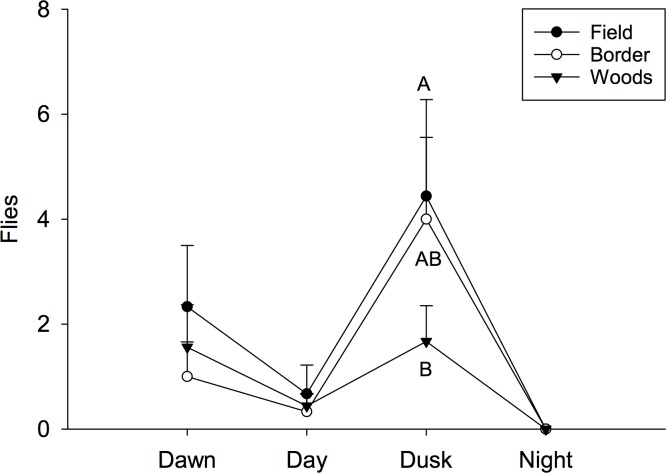
Mean number of spotted wing drosophila captured at different times of day and location near the orchard in 2016. Different letters signify statistical differences among locations within times of day (LSMEANS test).

In 2016, fewer than one-half the total number of flies per sample were captured compared to the previous year. Consequently, there was not a detectable time by habitat interaction in the 2016 data (F = 0.78; *df* = 6,96; P = 0.5887). Additionally, there was not a significant main effect for location (1.16; *df* = 2, 96; P = 0.3185), but there was a detectable difference among times of day (F = 8.70; *df* = 3, 96; P = < 0.0001). Captures started at 1.6 (± 0.5) flies at dawn and then decreased to 0.5 (± 0.2) flies during the day before peaking at 3.3 (± 0.8) flies at dusk; no flies were captured during the night (Fig 10). When examining at the sex ratio of captures flies, nearly twice as many female flies (94 females) were captured compared with males (53 males). No flies were captured during the night (Table 1). There was not a time of day by location effect (F = 1.30; *df* = 4, 28; P = 0.2943), but there were significant differences among locations (F = 3.62; *df* = 2, 28; P = 0.0401) and among times of day (F = 3.45; *df* = 2, 28; P = 0.0460). Sex ratio was clearly female biased during the day and in the field.

**Table 1:** Ratio of females to total fly captures.

Location	Dawn	Day	Dusk	Night
**Field**	1.00 (2)	1.00 (2)	0.66 (3)	0.00 (0)
**Border**	0.42 (7)	0.86 (7)	0.33 (2)	0.00 (0)
**Woods**	0.68 (6)	0.70 (4)	0.58 (4)	0.00 (0)

Ratio of females to total fly captures (n = number of data points with at least one fly) by time of day and field location. Ratio values above 0.5 indicate female bias, while values below 0.5 indicate male bias in the trap captures.

Mean relative humidity between times of the day was statistically significant (*F* = 434.7, *p* <0.0001, *df* = 3) for each 6-hour time period. Mean relative humidity for the night period (93.1 ± 0.60%) was highest, followed by dawn (88.5 ± 0.59%), dusk (78.2 ± 0.62%), and day (64.1 ± 0.60%), each period was significantly different from the others. Relative humidity was significantly different (*F* = 4.01, *p* = 0.0183, *df* = 2) between the three locations. The mean relative humidity was significantly higher in the woods (82.9 ± 0.62%) than at the border (80.7 ± 0.62%) and in the field (80.8 ± 0.62%). Temperatures were significantly (*F* = 5.09, *p* = 0.0062, *df* = 2) higher on the border (25.6 ± 0.25°C) than in the woods (24.9 ± 0.24°C). The mean temperature for the field (25.2 ± 0.26°C) was not statistically different from either the woods or border locations. Temperatures were significantly (*F* = 1735.8, *p* <0.0001, *df* = 3) different between times of the day. The highest mean temperature was the 6-hour day period (31.5 ± 0.20°C), followed by dusk (25.3 ± 0.18°C), dawn (23.6 ± 0.18°C), and night (21.2 ± 0.19°C).

## Discussion

Insects across all platforms of life make biologically-based decisions such as when to mate, search for a food, and when to be active based on the climate around them. Three of the main abiotic cues in the environment that drive insect decision making are temperature, humidity, and light, as well as the 24-hour oscillations between these cues [13, 26, 27, 28]. In the present study we assessed the activity of *D*. *suzukii* in unmanaged blueberry fields by setting up a series of weekly trapping routines designed to determine which times of day and locations in blueberry orchards *D*. *suzukii* are most active with respect to the temperature and relative humidity. There has been a great deal of work done with other insects regarding activity rhythms based on time of day. A study on the gold swift butterfly showed that reproductive activity is centered around the dawn hours due to the need to avoid mammalian predators and engage in morning mating [29], a similar pattern was observed in *D*. *suzukii* in the present study. While some insects and spiders find dawn to be optimal period for peak activity, other animals such as the table mountain stag beetle, Chinese rose beetle, and wolf spider are nocturnally active [30, 31, 32]. The results of the present study show that *D*. *suzukii* is least active at night and we hypothesize that it uses the nighttime as a period for sleep.

An important biological process in many insects and other animals is a circadian rhythm, displayed by increased activity between the time periods of dawn and dusk [33]. Insects that are most active during these periods are said to be crepuscular. Recent studies have shown that the crepuscular activity rhythms of *Lutzomyia longipalis* (Diptera: Psychodidae) and *Psorophora champerico (*Diptera: Culicidae), as well as *Pityophthorus juglandis* (Coleoptera: Scolytidae) are modulated by environmental stimuli such as temperature, photoperiod, and humidity [34, 35, 36]. Trap data show that in unmanaged blueberry orchards the activity of *D*. *suzukii* is bimodal and peaks during the crepuscular range of dawn and dusk.

The dawn and dusk periods are significant in terms of environmental stimuli due to the presence of twilight. The importance of twilight in adjusting the crepuscular activity patterns of *D*. *melanogaster* under laboratory conditions has been previously studied [37]. In the present study we correlate crepuscular activity to temperature and relative humidity that *D*. *suzukii* are exposed to during these dawn and dusk periods. The average temperature during dawn and dusk period for our study ranged from approximately 23–26°C across each location, indicating a possible ambient temperature that *D*. *suzukii* prefers in a field environment when compared to the average temperature during the day (31–32°C) and night (19–21°C) periods.

The question of where *D*. *suzukii* spends its time during the hottest part of the day, in relation to the blueberry bush and its canopy, remains largely unknown. There has been little work done to date on the effect of canopy on activity of agricultural pests, however, previous work on modeling temperature and humidity profiles under forest canopies [38] suggests the possibility that conditions underneath the canopy of blueberry bushes might support *D*. *suzukii* reproduction when conditions outside the canopy are normally unfavorable. We used the “Field Cage” and “Field Infestation” studies to address this question by measuring reproductive success of *D*. *suzukii* in a true field environment with respect to time of the day and location under the canopy of a blueberry bush. Previous studies on the effect of temperature and humidity on the oviposition and development of the codling moth (Lepidoptera: Tortricidae) and azuki bean weevil (Coleoptera: Bruchidae) show that oviposition site selection and development are influenced by orientation to the sun with respect to the increase in temperature within areas exposed to direct sunlight. Locations in the shade, associated with lower temperatures, were preferred over locations in direct sunlight. In direct sunlight conditions were not suitable for egg development and hatching [39, 40]. Results of the present study are consistent with these findings as significantly more eggs were laid per female on blueberries located inside the canopy of the bush.

Additionally, oviposition was significantly higher at night (including dawn and dusk periods) than during the day, consistent with recent studies which showed that eggs laid per female increase between the ambient temperature range of 25–28°C, which is similar to night temperatures in the field for this study [8, 41]. Pupation and adult eclosion were the same for both field trials in the present study, indicating that larvae which successfully pupated also reached adulthood. This could be due to the lack of competition for resources as a recent study shows that pupation and adult eclosion are affected by the quality and quantity of the host resources [42]. In the present study, such a small percentage of the eggs oviposited reached adulthood suggesting competition for resources was not a determining factor for successful larval development. The percentage of pupation and adult eclosion was significantly less during the day period (~2%) than the night (~18%) for the “Field Cage” experiment. Similarly, in the “Field Infestation” experiment the mean pupation and adult eclosion were 15% during the day and 25% during the night. These results are significantly lower than the observed values for *D*. *suzukii* kept under laboratory conditions (46–52%) which are consistent with the recent studies that show significant decreases in pupation and adult eclosion as temperatures approach upper developmental thresholds [28, 43, 44].

The results of the present study show that the number of adults per female was significantly higher in both field studies during the night period. Additionally, females located under the canopy during the day had significantly more progeny reach adulthood; this suggests the shade conferred by the canopy had a direct positive effect on reproduction. Previous literature shows that male *D*. *suzukii* could be sterile above 33°C. Based on those studies the significant differences observed in reproductive success in the day and night periods could be attributed to eggs laid by females being unfertilized due to male sterilization. Therefore, future studies should focus on investigating the effects of high temperature exposure on fertility of both male and female *D*. *suzukii*.

Temperature during the peak activity periods for *D*. *suzukii* was slightly lower in the woods and humidity was higher, this provides a possible explanation as to why fewer flies were trapped in the woods during peak activity hours. Additionally, our results indicate that relative humidity was a strong determinant of activity when viewed in terms of the interaction between location and time of day. Average relative humidity in the range of 80–90% was ideal for peak *D*. *suzukii* activity in the field and border locations during crepuscular hours. These findings are consistent with previous work done on insects found to be most active during crepuscular hours [30, 33, 34, 35, 36, 45, 46, 47].

For the “Field Cage” and “Field Infestation” studies, relative humidity was not positively correlated to the significance of day-night difference in pupation and oviposition. Instead we posit from the present study that the conditions formed as a result of the interaction between humidity and temperature at night create a climate conducive to *D*. *suzukii* reproduction. Supporting this finding is the fact that the night period included the dawn and dusk periods which have previously been shown to be the two bimodal activity peaks [16, 33, 36, 45, 46, 47].

Our study is the first attempt to document diel periodicity of *D*. *suzukii* under field conditions and elucidate the impact of temperature and relative humidity on *D*. *suzukii* daily activity and reproductive success in the field. These findings have practical implications for fruit producers contending with *D*. *suzukii* as a crop pest and will enable them to implement management strategies during the peak activity periods (dawn and dusk) in and around the field in order to maximize effectiveness. This will lead to fewer insecticide applications needed to control *D*. *suzukii*. However, in a laboratory-based study Hamby et al. (2013) showed that flies may be relatively less susceptible to insecticides due to increased activity of some of the detoxification enzymes during those peak activity periods [16]. Though it is very important to note that insecticide applications during periods of increased activity will lead to a larger number of *D*. *suzukii* being directly exposed to the insecticide treatment as opposed to the spray residues after treatment. A number of studies have shown that majority of the insecticides effective against *D*. *suzukii* have short residual efficacy ranging from 1–3 days after spray application [47]. Insecticide applications are normally not targeted for specific time periods but rather are carried out whenever time allows based on individual schedules for each grower (unpublished observation). Many times this results in insecticide applications during daytime hours or late morning when *D*. *suzukii* is no longer as active in the field. During this time *D*. *suzukii* activity enters an afternoon ‘siesta’ due to temperature and humidity being inhospitable.

## Conclusions

We conclude that *D*. *suzukii* reproductive success is greatly diminished during the day mainly due to harsh climatic conditions. The majority of reproductive activity occurs primarily during the crepuscular hours when climatic conditions including temperature and relative humidity as well as circadian rhythms favor both activity and reproduction. These results also suggest that the majority of *D*. *suzukii* successfully complete their development inside fruit that is fully shielded by the canopy of the blueberry bush where they are most likely to experience lower temperature and higher humidity. However, further investigation of *D*. *suzukii* chronobiology as it relates to reproductive success and susceptibility to management practices in the field should be the focus of future studies.

## Supporting information

S1 Table The supporting information files shows the average temperatures and relative humidity collected during the field cage study. (XLSX)

S2 Table The supporting information files shows the average temperatures and relative humidity collected during the field infestation study. (XLSX)

S3 Table The supporting information files shows the average temperatures collected during the time of day study for 2015. (XLSX)

S4 Table The supporting information file shows the average temperatures collected during the time of day study for 2016. (XLSX)
